# Women empowerment and minimum daily meal frequency among infants and young children in Ghana: analysis of Ghana demographic and health survey

**DOI:** 10.1186/s12889-021-11753-1

**Published:** 2021-09-17

**Authors:** Louis Kobina Dadzie, Joshua Amo-Adjei, Kobina Esia-Donkoh

**Affiliations:** Department of Population and Health, University of Cape Coast, Cape Coast, Ghana

**Keywords:** Minimum daily meal frequency, Infant, Young child, Feeding practice, Women empowerment

## Abstract

**Background:**

The nutritional quality of food has an important impact on the health and well-being of families, especially children whose bodies need to grow, develop and reach their full physical and mental potential. Traditionally, women in Ghana provide care and nourishment for their children and families if they have the means to do so or if they are financially, legally and socially empowered. Women’s empowerment is not only important for women’s human rights, but also improves nutrition and health outcomes of both mothers and their children. Women’s empowerment is concerned with increasing ability to make strategic life choices in situations where the ability was hitherto denied. This study sought to investigate the association between women’s empowerment and minimum daily meal frequency (minimum number of meals to be consumed in a day) in Ghana.

**Methods:**

The study used data from the 2014 Ghana Demographic and Health Survey (GDHS). A sample of 1640 mother-child dyad was used. Mothers ages ranged from 15 to 49 while children’s ages ranged from 6 to 23 months. Univariate and multiple linear regression techniques were applied to identify women empowerment (economic, socio-familial and legal) and sociodemographic factors associated with minimum daily meal frequency scores. Data was analyzed by the STATA statistical package software version 13.0. Statistical significance level was set at *P* < 0.10.

**Results:**

Data from decisions on large household purchases (β = 0.351, *p* < 0.01) family visits (β = 0.743, *p* < 0.01), home ownership (β = − 0.245, *p* < 0.10), age of child (β = 1.387, *p* < 0.01), mother’s educational attainment (β = 0.496, *p* < 0.10) and place of residence (β = − 0.298, *p* < 0.10) showed significant positive association with minimum daily meal frequency in Ghana.

**Conclusion:**

Minimum daily meal frequency was largely influenced by economic and socio-familial factors that contribute to empowerment of women. as decisions on large household purchases and family visits showed significant positive association with minimum daily meal frequency. Interventional programs should target households and mothers with lower socio-demographic characteristics such as lower educational levels and low economic status to improve minimal daily meal frequency in their children thereby ensuring better child health and well-being.

## Key messages

Minimum daily meal frequency is influenced by various dimensions of empowerment of women such as economic and socio-familial empowerment.

There is the need for improved advocacy for women to be involved in decision-making on family visits and large household purchases as these activities promote frequent meal attainment of children

Interventional programs should target households and mothers with lower socio-demographics characteristics such as lower educational level, lower income and those residing in rural areas to improve feeding at least the minimum daily meal frequency to children for their better health and well-being.

## Background

The nutritional quality of food has an important impact on the health and well-being of families, especially children whose bodies need to grow, develop and reach their full physical and mental potentials [1]. Accordingly, The Convention on the Rights of the Child recognizes that every infant and child have the right to good nutrition [2]. This right has also been expressed in the United Nations’ Sustainable Development Goal (SDGs). One of the SDGs (Goal #2) is tasked to “End hunger, achieve food security, improve nutrition and promote sustainable agriculture” while another SDG (Goal #3) should “Ensure healthy lives and promote wellbeing for all at all ages”.

Infant and young child feeding (IYCF) is considered a key area to improving child survival and promoting healthy growth and development [3–6]. Improved child nutrition is protective against several childhood illnesses, including gastrointestinal infections and malnutrition and fosters better development overall [7]. To implement these SDGs, the World Health Organisation (WHO) and the United Nations Children’s Fund (UNICEF) recommend the introduction of nutritionally adequate and safe complementary (solid or semi solid, or soft) foods for children from six months upwards together with continued breastfeeding up to two years of age or beyond [8]. Consequently, the WHO designed the IYCF tool with the view to improve and protect, promote, and support optimal infant and young child feeding [9]. One of such indicators is the minimum daily meal frequency which can be defined as minimum number of meals to be consumed by an infant or young child in a day [9–11].

Despite the immense benefits of children achieving minimum daily meal frequency, few children receive nutritionally adequate and safe complementary foods appropriate for their age [12]. Evidence shows that about 52% of all children 6–23 months of age are not receiving the minimum recommended number of meals per day with some countries in South Asia and other countries in sub-Saharan Africa having the lowest rates of minimum daily meal frequency of all countries studied [9]. In Ghana, only 43% of children within the age range of 6–23 months were fed with the minimum number of meals in a day [9, 13]. Many factors account for the inability of caregivers to provide adequate nutrition for children. These factors include household poverty, availability of food, personal and food insecurity, maternal ill health and stressful mealtimes [14].

Challenges associated with child feeding practices and undernutrition in Ghana results from poverty, lack of financial support from husbands or partners, cultural beliefs/ practices, excessive workload on the part of the caregivers and interference of grandmothers in the family food resource management [15–17]. Some scholars (e.g. Mulenga et al., 2019 [18]) contend that underlying these issues is the lack of women’s empowerment. Women and girls constantly face various forms of disempowerment in many aspects of life, including health, economic, social and political aspects, which to a to a large extent, create discriminatory tendencies and distortions in the human development pathways [19, 20]. In essence, women’s empowerment is not only important for women’s human rights, but also improves nutritional and health outcomes for both mothers and their children [21, 22].

Despite efforts of health workers to increase the number of children attaining the recommended feeding and health practices, not much success has been achieved. This is because, feeding practices are often difficult to change as they are directly related to varied economic, socio-cultural and religious factors in the community and to various dynamics prevailing at the household level [23]. While the study acknowledges that there has been an increasing and impressive scholarship on IYCF in Ghana, there was little focus on empowerment of women.

Women’s empowerment is of importance given that several development intervention programs have explicitly aimed at women’s empowerment [24]. Some of these programs have targeted micro-credit for women as an economic empowerment valve as well as formal education [25, 26]. Empowerment theory [27] assumes that personal, interpersonal and environmental resources are needed to increase and improve the skills, knowledge and motivation of people to achieve valid roles. They propose strategies for capacity building, awareness building and skill development to improve the status of the marginalized. This theory suggests that when women are denied access to resources needed for good health, interpersonal skills and valued social roles, they are rendered powerless and their functioning is undermined. The present study, therefore, sought to determine the association between women’s empowerment and minimum daily meal frequency of infant and young children in Ghana.

## Methods

### Source of data and sampling procedure

The study used data from the 2014 Ghana Demographic and Health Survey (GDHS). The GDHS is a cross-sectional nationwide survey designed and conducted every five years since 1988. The data was collected on fertility, antenatal care, delivery care and postnatal care, contraceptive use, child health, and family planning. The GDHS generally focuses on child and maternal health, and is designed to provide adequate data to monitor the population and health situation in Ghana. Data was also collected men at each administration of the survey to provide a context for understanding the health of women and children. The 2014 GDHS interviewed 9396 women aged 15–49 years from 12,831 households, covering 427 clusters throughout Ghana [13]. The 2014 GDHS followed a two-stage sample design. The first stage involved selecting sample points (clusters) consisting of enumeration areas (EAs) delineated for the 2010 Population and Housing Census (PHC). A total of 427 clusters were selected, 216 in urban areas and 211 in rural areas. The second stage involved the systematic sampling of households. A household listing operation was undertaken in all the selected EAs in January–March 2014, and households included in the survey were randomly selected from the list. About 30 households were selected from each cluster to constitute the total sample size of 12,831 households.

### Study population and sample selection

**Fig. 1:**
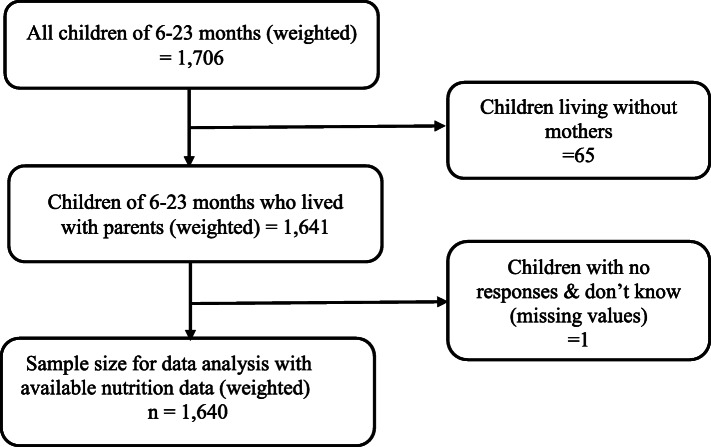
Study population and sample selection

Dyads of mothers (15–49 years) and children (6–23 months) were extracted from the women’s questionaaire and constituted the population for analysis. This study employed this group because it forms the basis of the WHO recommendation in calculating the IYCF indicators. The study sample (1640 children) was selected out of all (1740) children aged 6–23 months old. The sample selection is indicated in Fig. 1 below.

### Acquisition of Data

The data for the study was acquired online from the GDHS website ate https://dhsprogram.com. A registration form was completed and a brief proposal of the study indicating what the data set was going to be used for was sent. Then, an approval was given to download the dataset.

### Description and definition of variables

The study used minimum daily meal frequency as the dependent variable. This indicator for appropriate complementary feeding was created in accordance to the WHO guidelines [28]. The present study used minimum daily meal frequency as the dependent variable. Attainment of this minimum daily meal frequency is important in vulnerable infants and young children so as to prevent malnutrition, especially stunting and micronutrient deficiencies, morbidity in children and infants. Minimum daily meal frequency was described as minimum number of times in the previous day that children, 6–23 months of age, received solid, semi-solid or soft foods [28]. The minimum required frequency varied by child age and breast-feeding status [22]. Minimum daily meal frequency is twice for breastfed infants aged 6–8 months, three times for breastfed children aged 9–23 months, and four times for non-breastfed children aged 6–23 months”. In the Demographic and Health Survey (DHS) set of questions, one of it was for the respondent to state the number of times the child received solid, semi-solid or soft food in the past day. Three questions assessed the feeding frequency of infant formula, milk and yoghurt. These frequencies were used to calculate the number of milk feeds which were all found in the women’s questionnaire of the GDHS.

The independent variables for the study were: age of child, sex of child, age of mother, mother’s educational attainment, place of residence (rural/ urban), household wealth/economic status, number of children in the household who are aged less than five years, who controls women’s income, decision-making on large household purchases, mother’s occupation, decision-making regarding family visits, decision-making on own health, attitude to violence, ownership of house and ownership of land.

Three dimensions of women’s empowerment were used in this study: These were

(i) economic empowerment (control over women’s income, decision making on large household purchases, women’s ability to work outside the home)
(ii) socio-familial empowerment (decision making regarding family visits, women’s own health, and attitude towards domestic violence under five scenarios) and
(iii) legal empowerment (women’s judicial and legislative entitlements over land and over house ownership)

Age of child was recoded into 6–8 months, 9–11 months, 12–17 months and 18–23 months. Sex of the child was categorical (male/female). The number of children less than five years and mothers’ age were captured as a continuous variable in the multivariate analysis. Control over women’s income, decision-making on large household purchases, decision-making on family visits and own health were recoded as (a) respondent alone, (b) respondent and husband /partner, and (c) husband /partner alone. Mother’s occupation was recoded as (a) Not working, (b) Agricultural/labour and (c) White collar worker. Attitude towards violence comprised a blend of questions on all types of violence (on questions pertaining to beating justification when wife goes out without telling husband, neglects children, argues with husband, refuses to have sex with husband and burns the food) combined and the response variable was recoded as (a) Don’t know, (b) No and (c) Yes.

### Statistical analysis

Descriptive statistics were run to show the nature of independent variables. Multiple linear regression was applied to determine the association of the independent variables on the dependent variable. This was because our dependent variable (meal frequency) was continuous [29, 30]. Four models in total were estimated to demonstrate the associations between the various dimensions of women empowerment as well as socio-demographic factors and IYCF practices. Model 1 constituted the economic empowerment variables; control over women’s income, decision on large household purchases and maternal occupation. In Model 2, socio-familial variables; decision on family visits, decision on own health and attitude to violence were added to model 1. Model 3 now included the legal empowerment variables of ownership of house and land to the Model 2. The last model now included the demographic characteristics comprising; the age of child, sex of child, age of mother, mother’s educational attainment, place of residence, household wealth status and number of children aged less than five years living in the household. The data processing was accomplished using STATA statistical version 13.0 software. The statistical significance level was set at *P* < 0.10 which is unorthodox but important for improving practice [31].

## Results

### Background characteristics of respondents

More than half (52%) of the infant and young children were males. The highest percentage (34%) of the children were within the age range of 12 to 17 months. Mothers’ age categories were as follows: (a) 15–19 years (6%), (b) 40–44 years (6%) and 45–49 years (2%). Mothers within the age category of 25 to 29 years constituted the highest proportion (28%) of respondents. Most (55%) of the respondents were residents in rural areas. Few (4.4%) of the mothers were better educated (with post-secondary education) while about a quarter of the mothers (26.55%) had no education. The highest (50.30%) proportion of mothers had secondary education. With regards to economic status or level of wealth, the results showed that the highest proportion of the respondents who were designated as the poorest was 22.26% while the proportion of those designated as the richest quintile was 17.44% (Table 1).

**Table 1:** Background characteristics of the respondents, categorical variables (*n* = 1640)

Variable	Category	Frequency	Percent (%)
*Sex of child*	Male	851	51.88
	Female	789	48.12
*Age of mother (Years)*	15–19	95	5.81
	20–24	296	18.01
	25–29	466	28.4
	30–34	372	22.7
	35–39	281	17.13
	40–44	104	6.37
	45–49	26	1.58
*Place of residence*	Urban	739	45.09
	Rural	901	54.91
*Educational level*	No education	435	26.55
	Primary	308	18.75
	Secondary	825	50.3
	Higher	72	4.4
*Age of child (Months)*	6 to 8	309	18.87
	9 to 11	270	16.46
	12 to 17	563	34.35
	18 to 23	498	30.32
*Level of wealth designation*	Poorest	365	22.26
	Poorer	353	21.55
	Middle	303	18.45
	Richer	333	20.3
	Richest	286	17.44

Source; Computed for GDHS 2014.

### Association between women empowerment and minimum daily meal frequency

The findings show that women empowerment had significant association with minimum daily meal frequency. Children of mothers who made decisions with their partners concerning large household purchases were reported to have increased minimum daily meal frequency compared to those who had their partners/others make decisions (β = 0.351, *p* < 0.01). With respect to decisions on family visits, a positive association was found with meeting minimum daily meal frequency for children of children compared to those whose decisions were made by partners or other persons aside themselves (β = 0.743, *p* < 0.01).

The results further shows positive significant association with attaining minimum daily meal frequency and women’s disapproval of violence against women (β = 1.171, *p* < 0.10). Unexpectedly, mothers who jointly owned houses with their partners had their children unlikely to attain minimum daily meal frequency as compared to mothers who did not own houses (β = − 0.245, *p* < 0.10). This might be as a result of male dominance in the house or diversion of the income generated from renting into other ventures aside meals for the child. Land ownership showed varying levels of significance. For instance, a woman’s joint ownership of land had significant positive association with attainment of minimum daily meal frequency for children than their counterparts who did not own lands (β =0.470, *p* < 0.01). (Table 2) Probable reason could be that mothers who owned lands had access to meals from the land or could attain income from the land to feed children the daily meal frequency.

**Table 2:** Regression analysis of women empowerment and demographics on minimum daily meal frequency

Independent variables	Category	Model 1	Model2	Model 3	Model 4
Economic factors					
*Control over women’s income*	Respondent alone	−0.0528 [− 0.618,0.513]	−0.0953 [− 0.670,0.479]	− 0.152 [− 0.727,0.423]	−0.068 [− 0.595,0.459]
	Respondent and husband/partner	−0.0819 [− 0.671,0.507]	−0.029 [− 0.638,0.580]	−0.119 [− 0.729,0.491]	−0.1 [− 0.668,0.468]
	Husband/Partner or other alone	Ref	Ref	Ref	Ref
	Respondent alone	0.251 [−0.109,0.612]	0.118 [− 0.268,0.505]	0.0807 [− 0.305,0.466]	−0.186 [− 0.542,0.169]
	Respondent and husband/partner	0.118 [−0.123,0.360]	0.273* [− 0.00830,0.554]	0.275* [− 0.00675,0.556]	0.351*** [0.0851,0.617]
	Husband/Partner or other alone	Ref	Ref	Ref	Ref
*Mothers occupation*	Not working	Ref	Ref	Ref	Ref
	White collar	0.0859 [−0.143,0.315]	0.128 [− 0.111,0.367]	0.132 [− 0.106,0.371]	0.0543 [− 0.191,0.299]
	Agricultural/labour	Ref	Ref	Ref	Ref
Socio-familial empowerment					
*Family visit decision*	Respondent alone		0.514** [0.0792,0.949]	0.551** [0.116,0.986]	0.743*** [0.329,1.157]
	Respondent and husband/partner		0.248 [−0.152,0.648]	0.241 [− 0.159,0.641]	0.304 [− 0.0638,0.672]
	Husband/Partner or other alone		Ref	Ref	Ref
*Own health*	Respondent alone		−0.059 [− 0.418,0.300]	− 0.0424 [− 0.402,0.318]	− 0.0127 [− 0.343,0.318]
	Respondent and husband/partner		−0.141 [− 0.505,0.222]	−0.16 [− 0.527,0.207]	−0.238 [− 0.585,0.109]
	Husband/Partner or other alone		Ref	Ref	Ref
*Attitude towards violence*	Don’t know		Ref	Ref	Ref
	No		1.169* [− 0.114,2.451]	0.866 [− 0.422,2.155]	0.243 [− 0.978,1.464]
	Yes		1.458** [0.156,2.761]	1.171* [− 0.135,2.477]	0.467 [− 0.785,1.719]
Legal Empowerment					
*House*	Does not own			Ref	Ref
	Alone only			0.135 [−0.385,0.654]	0.118 [− 0.375,0.611]
	Jointly only			−0.345** [− 0.664,-0.0265]	−0.245* [− 0.535,0.0440]
	Both alone and jointly			−0.198 [− 0.885,0.489]	−0.357 [− 0.984,0.269]
*Land*	Does not own			Ref	Ref
	Alone only			−0.129 [−0.545,0.287]	− 0.358* [− 0.738,0.0221]
	Jointly only			0.579*** [0.239,0.919]	0.470*** [0.154,0.785]
	Both alone and jointly			0.191 [−0.509,0.891]	0.263 [−0.380,0.905]
Demographic factors					
*Age of child (Months)*	6 to 8				Ref
	9 to 11				1.179*** [0.781,1.577]
	12 to 17				0.646*** [0.361,0.932]
	18 to 23				1.387*** [1.094,1.681]
*Sex of child*	Male				Ref
	Female				−0.0885 [− 0.302,0.125]
*Mother’s age (Years)*					0.000872 [−0.0179,0.0196]
*Highest Educational level*	No education				Ref
	Primary				−0.325* [− 0.707,0.0574]
	Secondary				−0.172 [− 0.520,0.176]
	Higher				0.496* [−0.0170,1.008]
*Residence*	Urban				Ref
	Rural				− 0.298* [− 0.600,0.00450]
*Level of wealth designation*	Poorest				Ref
	Poorer				−0.0412 [−0.429,0.346]
	Middle				−0.163 [− 0.599,0.274]
	Richer				−0.0345 [− 0.513,0.444]
	Richest				−0.238 [− 0.808,0.332]
*Number of children less than 5 yrs*					−0.0163 [− 0.159,0.127]
_cons		3.407*** [2.834,3.980]	1.942*** [0.516,3.369]	2.306*** [0.866,3.747]	2.465*** [0.812,4.118]
R-sq		0.006	0.041	0.073	0.289
adj. *R*^2^		−0.004	0.016	0.034	0.231

90% confidence intervals in brackets; Ref = Reference; *p < 0.10, **p < 0.05, ***p < 0.01.

Model 1 constituted the economic empowerment variables.

Model 2 added the socio-familial variables;

Model 3 included the legal empowerment variables;

Model 4 added the demographic characteristics or variables.

Again, increasing child’s age (18–23 months) was positively associated (β = 1.387, *p* < 0.01) with meeting the required daily meal frequency. It is possible that older children can request for food or get food from family members and friends in the community to help them reach minimum daily meal frequency. The results also show a positive association between educational attainment of mothers and the minimum daily meal frequency of their children. Mothers with higher education (β = 0.496, *p* < 0.10) reported increased daily meal frequency for their children probably because of their better knowledge of the importance of good nutrition to the health of their children. A negative significant relationship with minimum daily meal frequency was found for women with primary education (β = − 0.325, *p* < 0.10) for the converse reason. Rural residence (β = − 0.298, *p* < 0.10) was negatively associated with attaining minimum daily meal frequency among children since women would not be in the known of the importance of feeding children with the minimum daily meal frequency (Table 2).

## Discussion

Understanding the drivers of mother’s ability to meet the minimum daily meal frequency among children less than two years is important for child survival and later life outcomes including education and earnings [32–34]. Anchored in empowerment thinking, this study aimed to understand the association between three indicators of women’s empowerment and minimum daily meal frequency among Ghanaian children aged 6–23 months. Data was pooled from the 2014 Ghana Demographic and Health Survey. The key findings are mother’s decision on family visits and decision on large household purchases had positive associations with meal frequency while, ownership of house had a negative association with meal frequency.

The first issue is that the decision on family visits had a significant association with the measure of minimum daily meal frequency. The argument is that as women gain more freedom to visit other families in the community, they tend to have wider social networks with other community members and exchange ideas and cultural beliefs on child-care practices [22]. Likewise, WHO (2020) [35] argues that mothers and families need support from community and mother support groups for their children to be fed optimally. Thus, the influence of social environment, close relatives and friends helps mothers to achieve the goal of providing appropriate meals for children [36, 37].

Decision-making control on large household purchases had significant positive association with minimum daily meal frequency. This finding is similar to studies in Philippines [38] and India [39], where maternal contribution to household income and power over household earnings and decision making were significantly associated with minimum daily meal frequency practices. This may be attributed to the fact that when women are involved in household purchases in large quantities, there is the likelihood to purchase food items. This further suggests that in order to meet the required number of meals a day for children, households need to purchase sufficient food stock [40–42].

Ownership of house decreased the ability of meeting IYCF practices for minimum daily meal frequency. In a study in sub-Saharan Africa [22], it was not quite clear why women’s entitlement to land and houses decreased their ability to feed children appropriately compared with similar women with no land or home ownership. This may be because asset inheritance rules can be complicated and also varies across settings [43]. However, in the context of India, Rao argued that granting land rights to women led to an increase of work burden without much improvement in their food security or social status [44].

Younger child age consistently showed no significance with meeting the criteria of minimum daily meal frequency. This finding is in line with previous studies in which younger children had higher odds of being fed inadequately in terms of frequency in Bangladesh [45]. Wondu et al., (2017) [46] also posit early child age bracket as risk group for inadequate minimum daily meal frequency. It has been noticed from the study that in the Ghanaian context, children are likely to meet this IYCF practice as they age which could be due to the fact that Ghanaian women practice IYCF better as the child survives. Similar results have also been noticed in Ethiopia [34, 47], Ghana [48] and Sri Lanka [49]. Evidence also suggest that children are introduced to more complementary meals as they grow and their teeth develops and hence, the frequency of meals is increased although there is a downside of loss of appetite which may lead to reduction in meal frequency [34, 50, 51].

Additionally, mother’s educational level had positive association with minimum daily meal frequency. Having high maternal education and interactions with family members and friends have been posited to enhance child nutrition [52, 53]. This validates previous work of Demilew et al., (2017) [54] which postulated that educated mothers might read books, leaflets and magazines, and might have a better chance of exposure to nutrition education about IYCF through mass media than their counterparts. Previous evidence in Nepal has it that mother’s education is vital for determining the feeding practices [55]. The probable reasoning could be that educated mothers are well informed and are more adherent to required feeding practices.

Rural settlement of respondents had a significant negative effect to meeting minimum daily meal frequency. This is inconsistent with an earlier finding which indicated that mothers who lived in urban areas were likely to attain the recommended minimum daily meal frequency [56]. They explain that urban mothers are aware and have more access to media which promotes complementary feeding practices. Although time restrictions, workload and other effects of modernization may affect mothers living at urban areas, mothers living in rural areas may not have enough education on complementary feeding practices altogether.

### Strengths and limitations

Limitation comes with self-reporting made by the mother. The GDHS data has a limitation of having only one day of diet recall per child, which may not be representative of the day-to-day dietary intake. Only one data point was used in the study because there have been changes in the definition of IYCF practices indicators by the World Health Organisation (WHO) and so comparison of indicators with previous years will be problematic. There is no loss to follow-up because participants are interviewed once. However, only associations and not causality can be inferred due to the cross-sectional nature of the data.

## Conclusion

The paper concludes that minimum daily meal frequency is influenced by various dimensions of empowerment of women such as economic and socio-familial empowerment. Specifically, joint decision on large household purchases, decision on family visits, ownership of land, age of child and mothers educational level improved minimum daily meal frequency. There is the need for improved advocacy for women to be involved in decision on family visits and large household purchases as it promotes frequent meal attainment of children. Additionally, mothers residing in rural areas impacted negatively on minimum daily meal frequency. Interventional programs by Ministry of Gender and Social protection, Ministry of Health as well as Ghana Health Service other stakeholders should target households and mothers with lower socio-demographics characteristics such as lower educational level to improve feeding at least the minimum frequency of children. Future studies could consider the influence of family members and husbands of mothers on minimum daily meal frequency.

## Data Availability

The dataset used in the study is freely available upon request from https://www.dhsprogram.com/data/dataset/Ghana_Standard-DHS_2014.cfm?flag=0

## Abbreviations

GDHS: Ghana Demographic and Health Survey
DHS: Demographic and Health Survey
SDGs: Sustainable Development Goals
IYCF: Infant and Young Child Feeding
WHO: World Health Organization
UNICEF: United Nations Children’s Fund
EAs: Enumeration Areas
PHC: Population and Housing Census
